# Autistic Disorder in Patients with Williams-Beuren Syndrome: A Reconsideration of the Williams-Beuren Syndrome Phenotype

**DOI:** 10.1371/journal.pone.0030778

**Published:** 2012-03-06

**Authors:** Sylvie Tordjman, George M. Anderson, Michel Botbol, Annick Toutain, Pierre Sarda, Michèle Carlier, Pascale Saugier-Veber, Clarisse Baumann, David Cohen, Céline Lagneaux, Anne-Claude Tabet, Alain Verloes

**Affiliations:** 1 Department of Child and Adolescent Psychiatry, Guillaume Regnier Hospital, University of Rennes 1, Rennes, France; 2 Laboratoire de la Psychologie de la Perception, Université Paris Descartes et UMR 8158 CNRS, Paris, France; 3 Child Study Center, Yale University School of Medicine, New Haven, Connecticut, United States of America; 4 Department of Genetics, Tours University Hospital, Tours, France; 5 Department of Genetics, Montpellier University Hospital, Montpellier, France; 6 Laboratoire de Psychologie Cognitive, Aix-Marseille Université, CNRS UMR 7290, and Institut Universitaire de France, Marseille, France; 7 Department of Genetics, Rouen University Hospital, Rouen, France; 8 Department of Child and Adolescent Psychiatry, Hospital Pitié-Salpétrière, University Paris 6, Paris, France; 9 Department of Genetics, AP-HP-Robert Debré University Hospital, Paris, France; Alexander Flemming Biomedical Sciences Research Center, Greece

## Abstract

**Background:**

Williams-Beuren syndrome (WBS), a rare developmental disorder caused by deletion of contiguous genes at 7q11.23, has been characterized by strengths in socialization (overfriendliness) and communication (excessive talkativeness). WBS has been often considered as the polar opposite behavioral phenotype to autism. Our objective was to better understand the range of phenotypic expression in WBS and the relationship between WBS and autistic disorder.

**Methodology:**

The study was conducted on 9 French individuals aged from 4 to 37 years old with autistic disorder associated with WBS. Behavioral assessments were performed using Autism Diagnostic Interview-Revised (ADI-R) and Autism Diagnostic Observation Schedule (ADOS) scales. Molecular characterization of the WBS critical region was performed by FISH.

**Findings:**

FISH analysis indicated that all 9 patients displayed the common WBS deletion. All 9 patients met ADI-R and ADOS diagnostic criteria for autism, displaying stereotypies and severe impairments in social interaction and communication (including the absence of expressive language). Additionally, patients showed improvement in social communication over time.

**Conclusions:**

The results indicate that comorbid autism and WBS is more frequent than expected and suggest that the common WBS deletion can result in a continuum of social communication impairment, ranging from excessive talkativeness and overfriendliness to absence of verbal language and poor social relationships. Appreciation of the possible co-occurrence of WBS and autism challenges the common view that WBS represents the opposite behavioral phenotype of autism, and might lead to improved recognition of WBS in individuals diagnosed with autism.

## Introduction

Williams-Beuren syndrome (WBS), first described in 1961 and 1962, is a rare genetic developmental disorder with a reported prevalence of between 1/7,500 and 1/25,000 live births [1]–[4]. Most cases are sporadic and until very recently the syndrome often went undiagnosed until adulthood. WBS syndrome is caused by an hemizygous deletion of contiguous genes on the long arm of chromosome 7 at 7q11.23 thought to arise from recombination between misaligned repeat sequences flanking the critical region during meiosis [5]–[7].

In 95% of affected individuals the size of the deletion is 1.55 Mb which encompasses about 25 to 30 genes [8]–[10], including the *elastin* gene (*ELN*) implicated in supravalvular aortic stenosis [11]–[13] and cutis laxa [14]. In 5% of affected individuals the size of the deletion is 1.84 Mb [15]. The specific contributions of most of the deleted genes to the cognitive and behavioral phenotype remains unknown [16]. For example, the contribution of the *LIM-kinase 1* gene to the specific cognitive profile is still controversial, with research teams reaching different conclusions [17]–[19]. It was recently suggested that two other genes (*GTF2IRD1* repeat domain-containing protein 1 and general transcription factor II-I *GTF21*) contribute to the specific cognitive deficit found in affected individuals [20]–[22]. The expression levels of the nonhemozygous flanking genes may be influenced by the deletion [23], further complicating efforts to clarify gene-behavior relationships.

The physical WBS phenotype includes a short stature, craniofacial abnormalities [24] with a characteristic dysmorphic face (elfin-like face), an odd gait, abnormalities affecting different systems such as the cardiovascular system (vascular stenosis), the musculoskeletal or endocrine systems, and frequent infantile hypercalcaemia leading to nephrocalcinosis [25], [26]. In addition, feeding problems (selective food refusal) and sleeping disturbances (such as night awakenings) have been reported in individuals with WBS [27], [28].

Patients with WBS are generally thought to display a typical heterogeneous cognitive and behavioral profile [29], [30] characterized by a mild to moderate intellectual disability associated with hyperacusis, severe impairments in visuo-spatial abilities and numeric processing, contrasting with relative good short term verbal memory, relative preservation of face processing [31], [32], great receptivity to music [33], and linguistic and social-affective skills. The latter abilities often produce a “cocktail party” personality including overfriendliness, lack of fear with strangers, strong pro-social compulsion [34], excessive talkativeness, and verbal fluency with extensive and expressive speech rich in vocabulary at least in adulthood [29], [35]–[37]. Domains of socialization and communication are considered to be relative strengths, whereas daily living and motor skills are relative weaknesses [30]. These strengths in social communication have led many to consider WBS as the opposite phenotype of autism.

Autism is defined in the DSM-IV-TR and the ICD-10 as a developmental disorder involving social and communication deficits and restricted interests/repetitive behavior with an onset prior 3 years old [38], [39]. Family and twin studies in autism have established the important role of genetics and indicate that autism is heterogenetic and polygenetic [40–42].

Six previous published articles have reported the co-existence of WBS and autistic disorder with severe impairments in communication and socialization. These studies, from four different countries, were limited by small sample sizes and were considered anecdotal: Turkey (N = 1) [43], United States (total N = 6) [44]–[46], Sweden (N = 4) [47], Germany (N = 2) [48]. Leyfer et al. [49] found 9 children who met autism spectrum criteria in a sample of 128 WBS children from United States (the sample included participants from the Klein-Tasman et al. Study [44]), but the paper was focused on the remaining 119 children and did not indicate precisely how many children met the diagnostic criteria for autistic disorder.

In the present study, we report on a sample of nine French individuals who had been diagnosed with autism and who were subsequently found on clinical and molecular genetic examination to have the common WBS deletion. The objective of the study was to better understand the range of phenotypic expression encountered in individuals with WBS and the relationships between WBS and autistic disorder. Findings were thought to have the potential to affect the general conception of WBS, to contribute to improved recognition of WBS in individuals diagnosed with autism, and, perhaps, to lead eventually to a more accurate estimation of WBS prevalence.

## Methods

### Patients

The study was conducted on nine French individuals with autistic disorder associated with WBS (five males and four females aged from 4 to 37 years old). All nine patients were attending care centers for autistic disorder (care centers in Chalons-en-Champagne, Troyes, Tours and Montpellier) and the identification of WBS was detected or confirmed in these patients by a network of French clinical geneticists (AV, CB, AT, PS), following a systematic clinical genetic examination. The clinical genetic examination showed typical WBS facial dysmorphism for all the 9 patients (hypertelorism, short nose with anteverted nostrils, thick lips with everted lower lip, and sagging cheeks) and a cardiac murmur for eight out of the nine patients associated with peripheral pulmonary stenosis or aortic stenosis.

Based on direct clinical observation of the patient by two independent child psychiatrists, a diagnosis of autistic disorder was made according to the criteria of DSM-IV-TR [38], ICD-10 and CFTMEA [39]. The diagnosis of autism was confirmed during this study by the Autism Diagnostic Interview-Revised (ADI-R [50]) and Autism Diagnostic Observation Schedule (ADOS [51]) ratings. Indeed, combining information from multiple sources results in a more consistent and rigorous application of autism diagnostic criteria based on the clinical judgment and the administration of the ADI-R competed by the ADOS [52].

Demographics, autistic behavioral domains and main physical features of the patients are presented in Table 1. Written informed consent was obtained from parents after explaining the study and its procedure to the parents and their children. The protocol was approved by institutional ethics review boards.

**Table 1:**
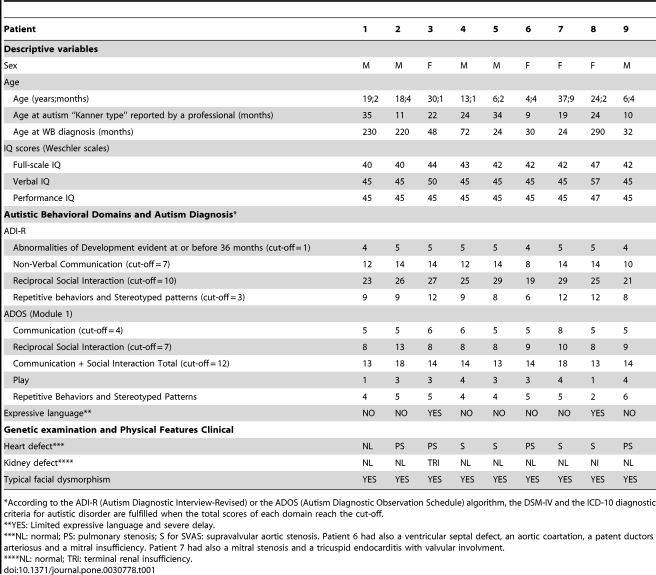
Demographics, autistic behavioral domains and main physical features of the patients.

* According to the ADI-R (Autism Diagnostic Interview-Revised) or the ADOS (Autism Diagnostic Observation Schedule) algorithm, the DSM-IV and the ICD-10 diagnostic criteria for autistic disorder are fulfilled when the total scores of each domain reach the cut-off.

** YES: Limited expressive language and severe delay.

*** NL: normal; PS: pulmonary stenosis; S for SVAS: supravalvular aortic stenosis. Patient 6 had also a ventricular septal defect, an aortic coartation, a patent ductors arteriosus and a mitral insufficiency. Patient 7 had also a mitral stenosis and a tricuspid endocarditis with valvular involvment.

**** NL: normal; TRI: terminal renal insufficiency.

### Behavioral and Cognitive Assessments

Cognitive functioning was assessed by two psychologists using the age-appropriate Wechsler intelligence scales (WPPSI-R, WISC, WAIS-III) and the Kaufman K-ABC [53]. All nine patients were severely cognitively impaired (mean full-scale IQ±SD: 42.4±2.1, with a range of 40–47).

Diagnostic and behavioral assessments were performed using the ADI-R scale and the ADOS scale. The ADI-R is an extensive semi-structured parental interview and the ADOS allows a direct observation of the patient through a standardized semi-structured situation of games. The ADI-R and the ADOS were administered by two trained psychiatrists certified in these scales administration. The ADI-R and ADOS scales assessed the three major domains of autistic impairments: reciprocal social interactions, verbal and non-verbal communication, stereotyped behaviors and restricted interests. We used the ADOS Module 1 that is administered to individuals with no language or beginning language skills (limited speech). The severity of impairments in the major domains and sub-domains of autism were scored using the subset of ADI-R or ADOS items included in the ADI-R or the ADOS algorithm. The individual ADI-R and ADOS items were scored on an ordinal scale from 0 to 3 according to autism severity; an item score of “0” means that the autistic behavior was not present. Interjudge reliability with respect to the critical distinction between mild, moderate and severe impairment was excellent, with an interjudge agreement of 94% observed between two expert raters. The ADI-R scale is validated to assess the behavior that was most abnormal during the 4 to 5 years-old period and the current behavior. The ADI-R algorithm is based on the 4–5 years-old period of life, but we reported also systematically the ratings for the current period of the subset of ADI-R items included in the ADI-R algorithm in order to see the evolution between these two periods of life (see Table 2). In addition, the ADI-R and the ADOS were coded independently but the ratings reported in Table 2 for the current period took into consideration the direct observation of the patient when a competency was observed by the trained psychiatrist during the ADOS administration. This approach, combining information from multiple sources based on the clinical judgment and the administration of the ADI-R completed by the ADOS, was undertaken in order to improve the confidence one can have in the current period assessment [52].

**Table 2:** Evolution of the autistic behavioral domains (social interaction, communication and stereotypies) and subdomains based on the ADI-R algorithm for the 4–5 years old period, and on the ADI-R algorithm completed by the ADOS algorithm for the current period.

Patient	1	2	3	4	5	6	7	8	9
Sex	M	M	F	M	M	F	F	F	M
Age (years;months)	19;2	18;4	39;1	13;1	6;7	4;4	37;9	24;2	6;4
**4 to 5 years old**									
Total Social Interaction	23	26	27	25	25	19	29	25	21
B1: failure to use nonverbal behaviors to regulate social interaction	5	6	5	5	6	2	6	6	4
B2: failure to develop peer relationships	7	8	6	8	8	7	8	7	7
B3: lack of shared enjoyment	6	6	6	4	6	3	6	6	4
B4: lack of socio-emotional reciprocity	5	6	10	8	9	7	9	6	6
Total Non-Verbal Communication	12	14	14	12	14	7	14	14	10
C1: delayed spoken language and failure to compensate through gesture	6	8	8	8	8	3	8	8	5
C4: lack of varied spontaneous make-believe or social imitative play	6	6	6	4	6	4	6	6	5
Total Repetitive Behaviors and Stereotyped Patterns	9	9	12	9	8	6	12	12	8
D1: encompassing preoccupation or circumscribed pattern of interest	4	4	4	4	4	2	4	4	2
D2: compulsive adherences to nonfunctional routines/rituals	2	1	4	1	2	1	4	4	2
D3: stereotyped and repetitive motor mannerisms	2	2	2	2	0	1	2	2	2
D4: preoccupations with part of objects or non-functional elements	1	2	2	2	4	2	2	2	2
**Current period**									
Total Social Interaction	13	19	17	17	17		21	15	16
B1: failure to use nonverbal behaviors to regulate social interaction	2	3	3	4	3		6	4	3
B2: failure to develop peer relationships	3	6	4	4	4		5	5	6
B3: lack of shared enjoyment	4	5	3	3	3		4	2	2
B4: lack of socio-emotional reciprocity	4	5	7	6	7		6	4	5
Total Non-Verbal Communication	7	11	10	10	9		12	7	8
C1: delayed spoken language and failure to compensate through gesture	4	7	6	6	5		7	7	4
C4: lack of varied spontaneous make-believe or social imitative play	3	4	4	4	4		5	0	4
Total Repetitive Behaviors and Stereotyped Patterns	8	8	10	7	8		11	4	8
D1: encompassing preoccupation or circumscribed pattern of interest	4	4	3	2	4		4	1	2
D2: compulsive adherences to nonfunctional routines/rituals	0	1	3	2	2		4	2	2
D3: stereotyped and repetitive motor mannerisms	2	2	2	2	0		2	0	2
D4: preoccupations with part of objects or non-functional elements	2	1	2	1	2		11	1	2

### DNA Isolation and Molecular Analysis

DNA was isolated from peripheral blood lymphocytes by standard procedures. Diagnosis of WBS was initially made with commercial FISH probes for the *ELN* gene locus on metaphase spreads. We excluded atypical deletion with quantitative multiplex PCR of short fluorescent fragments (QMPSF), a method based on the simultaneous amplification of multiple short exonic sequences using dye labeled primers under quantitative conditions [54]. Amplicon sequences where chosen in 6 genes from the common deletion (centromeric to telomeric: *FKBP6*, *BAZ1B*, *STX1A*, *ELN*, *LIMK1*, *CYLN2*, *GTF1*) and in the centromeric *CALN1* and telomeric *HIP1* genes that closely flank the common WBS deletion.

## Results

We present a thorough description of the 9 patients using detailed tables, descriptive narratives and summary descriptive statistics. Phenotypic aspects held in common with the typical autism phenotype are first described, then aspects held in common with the typical WBS presentation are presented. Finally, additional observed characteristics that are atypical of WBS are presented.

### Behavioral Phenotype in Common with Autism

All the patients fulfilled the ICD-10 and DSM-IV diagnostic criteria for autistic disorder based on the ADI-R (4–5 years old period) and ADOS (current period) algorithms (see Table 1). They all displayed during the 4–5 years old period severe impairments in the three main behavioral domains of autistic disorder (Communication, Reciprocal Social Interaction, Repetitive Behaviors and Stereotyped Patterns). In addition, autistic behaviors (social communication withdrawal including absence of direct gaze, social smiling, facial expressions and vocalization directed to others) were first noticed for all the patients in the first year of life by their parents, and before three years old they were all autistic according to Kanner criteria based on a professional judgment.

The five males with WBS showed an absence of verbal language whereas two out of the four girls were nonverbal but the two other girls displayed verbal language and even excessive talkativeness (mainly based on words or short utterances but with no daily use of adapted phrase speech including a verb). The two verbal females had an important delay in verbal language development and the first single words did not appear before 3.5 years-old. In addition, non verbal communication was also severely impaired for all our patients with a failure to compensate through gesture the lack of spoken language, and also a systematic lack of spontaneous make-believe play.

Reciprocal social interaction assessed by the ADI-R for the 4–5 years old period of life showed a severe lack of shared enjoyment, and also a failure to develop peer relationships. However, current period ADI-R social interaction scores were substantially improved for all the eight patients above 6 years old (for the 4.4 years old patient, the 4–5 years old period corresponds to the current period). This included a normalisation of their capacity of showing and directing attention for five patients and positive responses to other children's contact, with a decrease of the social autistic withdrawal and even the possibility to have a friend for three of them. Similarly, at the current period, the avoidance of direct gaze contact was not observed for three patients out of the nine individuals of the study (whereas it was found in all the patients except one girl during the 4–5 years age period), and the range as well as the appropriateness of facial expressions were normal or almost normal for five patients. The improvement in ADI-R assessed social interaction from the 4–5 years old period to the current period, was observed in all the patients above 6 years old and it appears, based on the ADI-R parental interview, that at least in some patients, it can occur as early as 6 years of age.

The ADI-R and ADOS assessments showed severe present and past stereotyped behaviors and patterns for the nine patients of our study. Indeed, all of them showed stereotyped behaviors such as hand flapping, clapping, fingers mannerisms and rocking. It is noteworthy that stereotyped behaviors can be observed in WBS individuals and are not specific of autism. In addition, rituals common in individuals with Williams Syndrome, such as lining up objects or sleep rituals, were also described for all of our patients. However, it may be of some interest to note that toe walk, a more characteristic autistic stereotyped behavior, was observed for five out of nine patients in our study. Also, quite striking was the existence in all nine patients, at the present period and during the 4–5 years age period, of spinning behaviors with fascination for spinning objects (plates, pens, keys, toy-car wheels or bus wheels, tops, balls, washing machine, electric fan, revolving door…), and their propensity for turning pages of books and magazines.

In addition, repetitive unusual sensory interests were present for the nine patients: preoccupation with tactile stimuli including warm water (running water, shower, swimming pool,), sniffing, licking, actively seeking “wind sensations”(manual or electric fan). Also, interests in musical objects or toys were systematically found, which could be relevant of their interest in music in general and their musical vocalization (vocalization following a melody or a song). Finally, self-injurious behaviors were observed such as slapping one's ear, biting one's hand, self-scratching until bleeding, elbow-banging, pulling off one's skin around the nails.

### Behavioral, Cognitive and Physical Phenotypes Typical of WBS

A sensory interest for music was observed in the nine patients of the study which is consistent with the good receptivity to music usually found in individuals with WBS and described in the introduction. Similarly, all the patients except one boy showed also hyperacusis to loud sounds, a typical feature of WBS. They covered their ears at screams, babies crying, sounds of jack hammer or of several people talking. The typical physical WBS phenotype was observed including a characteristic dysmorphic face (elfin-like face) in all the 9 patients and heart defects in eight out of the nine patients. (see Table 1). Furthermore, the nine patients had balance disturbances with an unstable walk and a common odd gait (head forward and bent back). Finally, sleep and feeding problems were reported. Indeed, six out of the nine patients had perinatal feeding disturbances, especially regurgitations or sucking problems, and two patients displayed selective food refusal, an eating problem often observed in WBS. More intriguing were the sleep problems: past and present sleep disturbances were reported for all our nine patients, based on sleep questionnaires and Actiwatch measures, including longer sleep latency associated with body rocking in the bed or clapping hands, as well as nocturnal and early morning awakenings. These sleep disturbances appeared during the first year of life and are still present for all of them.

### Behavioral, Cognitive and Physical Phenotypes Atypical of WBS

In a number of ways, our nine patients did not fit the behavioral, cognitive and physical profiles that have been generally considered to be typical of individuals with WBS. Indeed, their verbal communication and social interaction impairments are not concordant with the high abilities in social communication (such as excessive talkativeness and overfriendliness) usually reported in WBS and described in the introduction. In addition, pointing appeared before the development of verbal language for all our patients (except one boy who did not point at all, but was also non-verbal), even if there was a delay compared to normal development. Pointing usually appears after and not before verbal language in individuals with WBS, which is considered to be a developmental particularity of WBS [55]. Moreover, all nine patients of our study showed a severe and homogenous intellectual disability. In particular, their Verbal IQ score was as low as their Performance IQ and full-scale IQ (IQ scores were at their lowest levels on the Weschler scales). Finally, a delay in height development is usually observed in WBS leading to a short adult height. However, although a delay in height development was noted during childhood, an average height was reached during adulthood for our post-pubertal patients.

### Genetic Results

In all the nine patients, the common WBS deletion at chromosomal band 7q11.23 was found. This included deletion of the WBS critical region (WBCR). Molecular characterization by FISH indicated that all the nine patients were deleted for the elastin gene.

## Discussion

### Main Findings

The nine individuals with WBS associated with autistic disorder studied had severe impairments in verbal communication (absence of spoken language for all the males and for two females, and severe delay in language development for the two other females), deficits in social interactions with a positive evolution in childhood, and pronounced stereotyped behaviors such as toe walk and fascination for spinning objects. The severe impairments in verbal communication and social interaction as well as the severe homogenous intellectual disability are contrasting with the typical features usually described in individuals with WBS (talkativeness, overfriendliness, mild to moderate intellectual disability with higher IQ scores on the verbal scale than on the perceptual performance scale [56]). However, severe impairments in social communication were also reported in the 6 previous case studies of WBS associated with autistic disorder [43]–[48]. It is noteworthy that talkativeness which is typical of WBS was present for the two verbal girls of our study, but not for any of the boys. The same observation was reported by Gillberg and Rasmussen [47] who noted that both autistic girls with WBS of his study showed talkativeness whereas the two boys were non verbal. This male prevalence of absence of verbal language in individuals with WBS associated with autistic disorder could be of interest with regard to the male prevalence of autistic disorder (4–5 times more males than females [57]). Similarly, characteristic autistic stereotypies, such as toe walk or fascination for spinning objects, were observed in the previous case studies of WBS associated with autistic disorder [46]–[48]. In addition, the nine patients showed sleep disorders (longer sleep latency, nocturnal and early morning awakenings) which are in line with the case studies of WBS associated with autism [46], [47], but appear more specific and frequent than what has been reported in WBS (sleeping difficulties were found in 45.5% of the WBS sample by Udwin and Yule [28]). Furthermore, longer sleep latency and nocturnal or early awakenings have been often reported in autistic disorder [58], suggesting that these sleep disorders are related to autism.

### Social Communication Deficits in WBS

Our findings raise the issue of social communication impairments in typical WBS with a continuum ranging from excessive talkativeness and overfriendliness to absence of verbal language and poor social relationships. Several recent studies report social communication impairments in individuals displaying the typical deletion of the WBS region, suggesting that social communication impairments might be underestimated in WBS [44], [59]–[61]. Indeed, recent studies in individuals with WBS suggest that language development does not follow an entirely normal pathway [55], [61]–[65] and might be delayed (for review, see [66]). In fact, linguistic abilities of children with WBS are not above their cognitive level and all aspects of WBS language (including vocabular acquisition and grammatical abilities) show delay and/or deviance throughout development [37], [62], [63], [67]. Although expansive and expressive speech rich in vocabulary are usually reported [68], case studies of WBS described by Stojanovik et al. [69] showed deficits in performance on standardized tests of expressive and receptive language. Furthermore, verbal language in WBS individuals appears not to be socially adapted and relevant to social communication. In fact, social communication appears to be impaired in WBS and real strengths in this domain can be questioned. For example, some studies indicated evidence for pragmatic language impairment including excessive chatter, conversations not well tuned to the partner as well as talking to themselves, socially inappropriate utterances and questions with also inappropriate initiation of conversation and stereotyped sentences [28], [61], [69]–[71]. At this point, it appears important to distinguish productive speech which can be preserved in WBS to verbal communication which is impaired often in individuals with WBS. It is noteworthy that these speech particularities can also be observed in individuals with autistic disorder. Some clinicians even argue that a diagnosis of high functioning autism could be applied to children with semantic-pragmatic disorder [72], [73].

Impairments in the social interaction domain, involving in particular poor social relationships, have been also described in WBS [44], . Thus, Davies et al. [70] reported that 96% of parents and caregivers of adults with WBS described problems with establishing friendships, and a large proportion also described other social difficulties, such as disinhibition and social isolation.

Finally, Laws and Bishop's study [61] suggests that the three autistic behavioral domains are impaired in WBS (social interaction, communication, stereotypies). Indeed, WBS patients can exhibit social communication impairments previously described, but also restricted and stereotyped behaviors or interests, such as hypersensitivity to sounds, picky eating, inflexibility, ritualism, obsessive and perseverating attitudes [60]. Social communication impairments and stereotyped behaviors seemed to be relatively common in WBS and this adds to the potential for WBS patients to be diagnosed with autism. Laws and Bishop [61] concluded that far from representing the polar opposite of autism, as suggested by some researchers [76], WBS would seem to share many of the characteristics of autistic disorder. More severe autistic behaviors, as observed in our sample, may be more common in WBS than reflected by the literature. It appears important to be aware of the behavioral phenotypic heterogeneity of WBS, especially for impairments in the social communication domain where a continuum ranging from mild to more severe impairments may be observed, in order to not misdiagnose individuals with WBS and associated autistic behavior.

### Subtypes of Autistic Disorder

Our study suggests the existence of different subtypes of autistic disorder based on characteristic behaviors and developmental profile.

Indeed none of our 9 patients displayed currently the autistic withdrawal described by Kanner [77] as a social withdrawal and commonly observed in individuals with autistic disorder. They showed actually, according to the direct observation of the patient or the parental interview, a social motivation with a desire and eagerness to interact with others, which was not the case at 4–5 years old. This is in line with the typical features of individuals with WBS who are usually described as highly sociable, empathic, friendly and warm [27], [44], [48], [78]–[82]. Social motivation assessment might be informative and useful to distinguish different subtypes of autistic disorder. It is noteworthy that social motivation has been found to be a very important quantitative trait related to autism [83]. Sung et al.. 's study [83] suggests that social motivation has a genetic basis and may be most promising for future gene mapping and for extending pedigrees by phenotyping relatives. Similarly, the avoidance of direct gaze contact, a characteristic autistic behavior, was not observed at the current period for three patients, suggesting that this behavior might be important to distinguish different subtypes of autistic disorder.

In contrast, all 9 patients of our study still showed, at the present period as well during the 4–5 years age period, spinning behaviors or intense interest for spinning objects which are characteristic autistic stereotyped behaviors but are not found in all individuals with autistic disorder.

Finally, as shown in Table 2, a developmental trajectory of social communication impairments appears in the WBS patients of our study involving improvement with time, observed especially after 6 years-old, in social interaction and nonverbal communication for all the eight patients above 6 years old, and improvement in verbal communication for two patients at 3.5 years-old. It is noteworthy that social interaction has been reported to discriminate children with autism and children with WBS [45]. This suggests, in line with Karmiloff-Smith with regard to developmental disorders [84], that it might be important to have a developmental approach taking into consideration the onset of autistic behaviors prior to age 3 years, but also the evolution throughout the lifespan and especially after 6 years-old.

### Genetic Perspectives

Future genetic research is required to ascertain the mechanisms underlying this association between WBS and severe autistic disorder, and to better understand the presence of such behavioral and cognitive profiles usually not reported in individuals with WBS. In contrast with excessive talkativeness typically reported in the common WBS deletion, speech delay has been observed in the duplication of the WBS region [85]–[88]. Furthermore, duplications of the WBS region have been recently found to be associated with severe delay in expressive language, mild to moderate intellectual disability and autism spectrum disorders, suggesting that specific genes within the WBS region can influence language and social development through gene dosage effects [85]–[89]. In this study, all 9 patients with WBS associated with severe autistic disorder, including severe expressive language delay, showed the common WBS deletion, which suggests the existence of other mechanisms than gene dosage effects at 7q11.23 on language and social development. Our findings suggest that specific genes of the WBS region influence social communication, and their expression may vary depending on genetic background interaction with other genes (such as the serotonin transporter gene that has been reported to modify the severity of the social and communication impairments in autism [90], [91]) and/or environmental factors (such as hyperserotonemia, given the well-replicated hyperserotonemia of autism [92] and previous reports of hyperserotonemia in WBS associated with autism but not in WBS without autism, [46], [93]).

### Limitations

The study necessarily used a sample of convenience and therefore can not make definitive statements about the prevalence of WBS in individuals with autism nor concerning the rate of occurrence of autism-related behavior in WBS patients. The use of the retrospective ADI-R assessment, although well-validated, applied to adult patients in order to ascertain their behavior at 4–5 years of age has limitations due to inaccuracy and bias of recall. However, the ADOS scale that allows to assess the present period and to give a current diagnosis of autism, confirmed the diagnosis of autistic disorder for all 9 patients (see Table 1).

### Conclusions

Our results suggest that comorbid autism and WBS occur more frequently than expected and that WBS should be excluded when considering possible single gene causation in individuals with autism. An underestimation of the co-occurrence of these two disorders might contribute to maintain the common view that WBS represents the opposite behavioral phenotype to autism. This could, in turn, contribute to the under-recognition of WBS and the underestimation of its prevalence. Similarly, this could contribute to the underestimation of autism in WBS.

Search for behaviors related to autism spectrum disorders in patients with WBS may have important clinical implications considering that some children with WBS could benefit from therapies offered to children with autistic disorder. Inversely, as underlined by Miles and Hillman [94], it is important for practitioners working with children with autistic disorder to ask systematically for a clinical genetic examination with a clinical morphology examination searching for genetic disorders associated with autism, including WBS.
